# Impact of DNA Repair Kinetics and Dose Rate on RBE Predictions in the UNIVERSE

**DOI:** 10.3390/ijms23116268

**Published:** 2022-06-03

**Authors:** Hans Liew, Stewart Mein, Thomas Tessonnier, Christian P. Karger, Amir Abdollahi, Jürgen Debus, Ivana Dokic, Andrea Mairani

**Affiliations:** 1 Clinical Cooperation Unit Radiation Oncology, German Cancer Research Center (DKFZ), 69120 Heidelberg, Germany; h.liew@dkfz-heidelberg.de (H.L.); juergen.debus@med.uni-heidelberg.de (J.D.); 2 Division of Molecular and Translational Radiation Oncology, National Center for Tumor Diseases (NCT), Heidelberg University Hospital, 69120 Heidelberg, Germany; s.mein@dkfz-heidelberg.de (S.M.); a.amir@dkfz-heidelberg.de (A.A.); i.dokic@dkfz-heidelberg.de (I.D.); 3 Heidelberg Institute of Radiation Oncology (HIRO), German Cancer Research Center (DKFZ), 69120 Heidelberg, Germany; c.karger@dkfz-heidelberg.de; 4 German Cancer Consortium (DKTK), 69120 Heidelberg, Germany; 5 Heidelberg Ion-Beam Therapy Center (HIT), 69120 Heidelberg, Germany; thomas.tessonnier@med.uni-heidelberg.de; 6 Faculty of Physics and Astronomy, Heidelberg University, 69120 Heidelberg, Germany; 7 Department of Medical Physics in Radiation Oncology, German Cancer Research Center (DKFZ), 69120 Heidelberg, Germany

**Keywords:** ionizing radiation, ion beam therapy, UNIVERSE, dose rate, DNA repair, modeling, RBE, rat spinal cord

## Abstract

Accurate knowledge of the relative biological effectiveness (RBE) and its dependencies is crucial to support modern ion beam therapy and its further development. However, the influence of different dose rates of the reference radiation and ion beam are rarely considered. The ion beam RBE-model within our “UNIfied and VERSatile bio response Engine” (UNIVERSE) is extended by including DNA damage repair kinetics to investigate the impact of dose-rate effects on the predicted RBE. It was found that dose-rate effects increase with dose and biological effects saturate at high dose-rates, which is consistent with data- and model-based studies in the literature. In a comparison with RBE measurements from a high dose in-vivo study, the predictions of the presented modification were found to be improved in comparison to the previous version of UNIVERSE and existing clinical approaches that disregard dose-rate effects. Consequently, DNA repair kinetics and the different dose rates applied by the reference and ion beams might need to be considered in biophysical models to accurately predict the RBE. Additionally, this study marks an important step in the further development of UNIVERSE, extending its capabilities in giving theoretical guidance to support progress in ion beam therapy.

## 1. Introduction

The favorable dose distribution and increased relative biological effectiveness (RBE) of ion beams in comparison to conventional radiation sources has driven their increasing adoption in radiotherapy. The RBE is defined as the ratio of doses applied by a reference radiation (normally photons) and an ion beam of interest to achieve the same biological effect and serves a central role as a metric in both radiobiology and radiation therapy. In clinical practice, a constant RBE of 1.1 for proton beams has been widely accepted as a sensible approximation. However, the proton RBE has been found to be dependent on several factors, most prominently the dose and linear energy transfer (LET) of the particles, with experimental evidence and biophysical models consistently implying an increase of RBE with LET [1,2]. As part of the world’s first clinical application of a raster scanned helium ion beam at the Heidelberg Ion-beam Therapy Center (HIT) in July 2021, treatment plans were based on RBE predictions from the “modified microdosimetric kinetic model” (mMKM) [3]. The model was previously tuned [4] and benchmarked [5] using extensive in-vitro data on the biological effect of helium beams as a function of dose and LET. The “UNIfied and VERSatile bio-response Engine” (UNIVERSE) is a mechanistic modeling framework being developed with the goal of enabling the description of clinically relevant modifying factors of radiation action for both conventional and ion beam irradiations [6,7,8,9,10]. It is supposed to help interpret pre-clinical studies, support the development of innovative radiotherapy concepts, and assess the necessity as well as implications of including additional parameters into clinical treatment planning. For example, the ion beam model of the framework was recently established [9], including a description of the effect of interference on the DNA damage repair process, which is consistent with its earlier published implementation for conventional radiation sources [6]. This allowed for the investigation of the effect of such a modifier of radiation action on the predicted RBE and its meaning for clinical scenarios.

Experimental investigations of the effect of the applied dose rates of both radiation modalities on the RBE of ion beams remain limited, although they are motivated by several applications, including the effect of bursts of cosmic radiation in spaceflight [11], the application of increased dose rates to avoid respiratory gating in radiotherapy [12], and investigations of the differential effects of laser driven ion beams [13,14,15]. Historically, dose-rate effects were rationalized using concepts such as the recovery of a finite repair capacity of the cell or its repair of sub-lesions [16]. The latter idea is followed to some extent by extensions of modern mechanistically motivated ion beam models, which include repair kinetics of DNA lesions to allow predictions of the effect of the dose rate [17,18,19,20,21]. In line with the sparse experimental data [12], studies of clinical scenarios based on these models predict the limited impact of the dose rate at lower doses, which are applied in conventionally fractionated ion beam therapy (~2 Gy) but expected a substantial loss of effectiveness in protracted irradiations at high doses, which are typical of hypofractionated approaches [17,18,19,20,21]. However, a study based on a more phenomenological modeling approach suggests that even at doses as low as 2 Gy, dose-rate effects could lead to local inhomogeneities in the effectiveness of actively scanned proton beams if the irradiation time is significantly prolonged [22]. While they were benchmarked using data from a split-dose experiment with a single dose and energy setting [17], none of the mentioned dose-rate extensions of mechanistic ion beam models were benchmarked against any measurements in clinically relevant fields (e.g., actively scanned SOBPs) or at high doses commonly applied in preclinical in-vivo experiments [17,20,21].

In the study presented here, the ion beam model of UNIVERSE is combined with a description of DNA damage repair kinetics that considers the clustering of damages, which has been successfully established in an earlier publication to describe dose-rate effects for sparsely ionizing radiation [8]. Applying this new model, we now investigate the impact of damage repair on the response to ion beams—as well as on the reference radiation—and thus on the RBE predictions within UNIVERSE and whether the changes are consistent with the trends described in the literature. Ultimately, predictions of the modified UNIVERSE, its previous version, and the existing clinical approaches are compared to RBE data obtained in rat spinal cord irradiated with high doses in a proton and helium spread-out Bragg peak (SOBP) by Saager et al. [23] and Hintz et al. [24], respectively. The benchmark will allow us to assess the necessity for biophysical models to consider dose-rate effects to accurately predict clinically relevant high dose in-vivo measurements and judge the ability of UNIVERSE to include another key parameter to its predictions, thereby increasing its potential to give theoretical guidance in pre-clinical studies.

In this work, only dose rates applied in conventional clinical ion beams are considered, thus potential tissue sparing effects that might arise at ultra-high dose rates are not considered within the presented model.

## 2. Results

**Figure 1:**
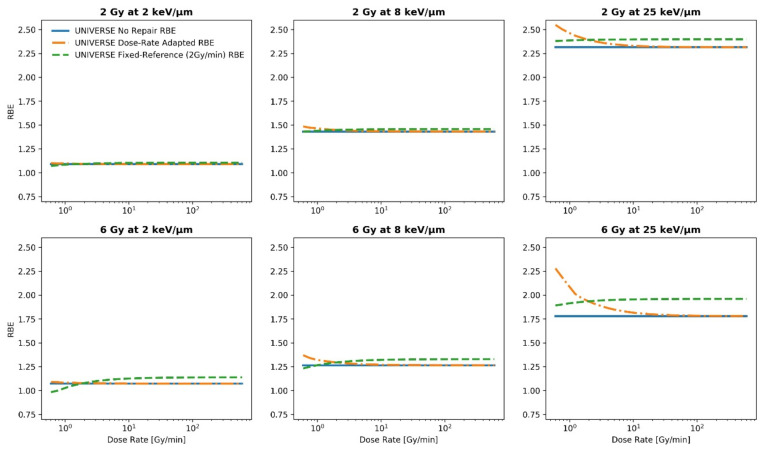
Proton RBE predictions for the DU145 cell-line over the applied dose rate for the fixed-reference RBE (2 Gy/min; green dashed line) as well as the dose-rate adapted RBE (dotted orange line) by using the extended version of the UNIVERSE for three representative LET values at moderate doses. The no-repair RBE is shown for reference (solid blue line).

**Figure 2:**
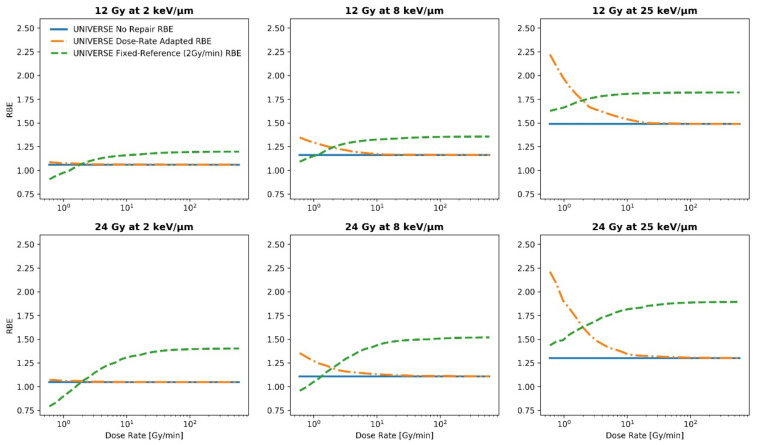
Proton RBE predictions for the DU145 cell-line over the applied dose rate for the fixed-reference RBE (2 Gy/min; green dashed line) as well as the dose-rate adapted RBE (dotted orange line) by using the extended version of the UNIVERSE for three representative LET values at higher doses. The no-repair RBE is shown for reference (solid blue line).

To study the general impact of introducing DNA repair kinetics and the applied dose rate into the UNIVERSE, the proton RBE was simulated over the range of 0.1 to 10 Gy/s at 2, 6, 12, and 24 Gy, each at 2, 8, and 25 keV/µm (Figure 1 and Figure 2). For each setting, the proton RBE was calculated with respect to a sparsely ionizing reference radiation at a fixed dose rate (2 Gy/min; green dashed line) as well as at the same dose rate as the proton irradiation (dotted orange line) by using the extended version of the UNIVERSE. To improve readability, the two definitions will be referred to as “fixed-reference RBE” and “dose-rate adapted RBE” from here on. The fixed-reference RBE is meant to represent the situation in most RBE studies and measurements, where the dose rate of the reference radiation is often a constant value. Here, a value of 2 Gy/min for the dose rate of the reference radiation was chosen as a representative value. On the other hand, the dose-rate adapted RBE shows the “intrinsic” efficacy of the protons in comparison to the reference radiation for a certain cell-line at the same dose rate for protons and photons. As a reference, the proton RBE prediction, which disregards any DNA repair kinetics and applied dose rates for both the reference radiation and the proton beam, is shown as well (solid blue line) and will be referred to as “no-repair RBE”. (For an overview of the RBE definitions please refer to Section 5.4). For demonstration purposes, the model parameters were set to represent the DU145 cell-line line as obtained from Adrian et al. [25] and El-Awady et al. [26], which has been used in a similar analysis in one of our previous publications [8] (Table 1). The fixed-reference RBE (green dashed line) is found to initially increase and then saturate towards high dose rates. The trend slightly weakens with increasing LET. On the contrary, the dose-rate adapted RBE (orange dotted line) initially decreases before saturating at a lower level, which is identical to the no-repair RBE (sold blue line). For the dose-rate adapted RBE, the trend is more pronounced for the higher LET settings. For both definitions, the range of RBE covered within the studied dose-rate ranges visibly increases with the applied dose. The same general trends of the fixed-reference and dose-rate adapted RBE were found for helium ions (see Supplementary Material Figure S1).

**Table 1:** Endpoint dependent UNIVERSE parameters applied in this work.

Endpoint	$K_{iDSB}$	$K_{cDSB}$	$T_{iDSB}^{1/2}\;[\min]$	$T_{cDSB}^{1/2}\;[\min]$	Reference
DU145	5.9 × 10^−3^	0.17	4	100	[25,26]
Rat Spinal Cord (considering repair during parameter fit)	3.5 × 10^−5^	9.8 × 10^−3^	11.4	129.6	[27,28,29]
Rat Spinal Cord (neglecting repair during parameter fit)	6.5 × 10^−3^	8.5 × 10^−3^	-	-	[27,28]

In order to further examine the possible discrepancy between the no-repair RBE (blue solid line) and fixed-reference RBE for the representative setup above, the relative difference between the two predictions for each presented setting are shown in Table 2. The relative difference between the predicted RBE values appear to generally increase over both dose and LET.

**Figure 3:**
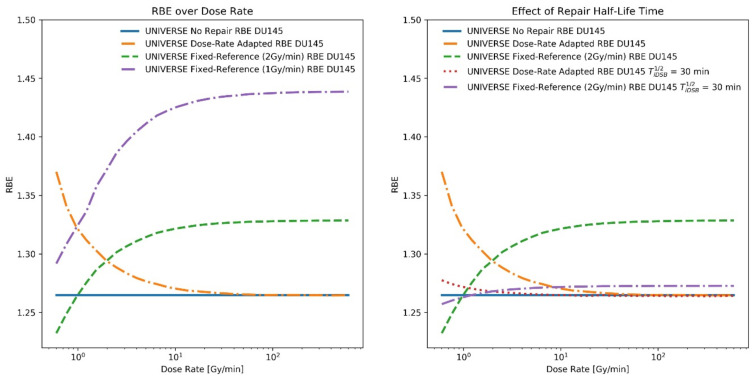
Proton RBE predictions for the DU145 cell-line over the applied dose rate for the fixed-reference RBE (2 Gy/min; green dashed line) as well as the dose-rate adapted RBE (dotted orange line) at 6 Gy and 8 kev/µm. The predicted no-repair RBE is shown for reference (solid lines). **Left** panel: An additional prediction for the same cell-line is shown, calculated based on a reference radiation source with a reduced fixed dose rate (1 Gy/min). **Right** panel: Additional predictions are shown for the same cell-line but a larger value for $T_{iDSB}^{1/2}$ (=30 min).

To illustrate the potential impact of the dose rate applied by the reference radiation on the predicted RBE, the fixed-reference RBE is plotted using two representative reference dose rates at the setting of 6 Gy and 8 keV/µm (values chosen for demonstrative purposes), as shown in the left panel of Figure 3. Shifting the dose rate of the reference radiation from 2 Gy/min to 1 Gy/min resulted in an increase of 8.3% in the predicted maximum RBE at the applied settings. Similarly, the proton RBE was recalculated using a longer representative repair half-life time for isolated damages $T_{iDSB}^{1/2}$ of 30 min to showcase the effect of this parameter on model predictions (right panel Figure 3). The dose-rate effect is visibly reduced when the longer repair half-life time is applied. The maximum value of the fixed reference RBE decreased by 4.2% and its maximum deviation from the no-repair RBE decreased from 5.5% to 0.6%.

As the effects of the applied dose rate on the RBE are progressively pronounced with an increasing dose, we examined whether the predictions of the modified UNIVERSE are consistent with the experimental data obtained using proton and helium beams applying high doses with distinct dose rates from those applied by the reference radiation. For this purpose, the rat spinal cord (RSC) studies by Saager et al. [23] and Hintz et al. [24] were used. These studies measured the RBE based on the *TD_50_*-values (dose at 50% effect probability) for the clinical endpoint of paresis grade II that was detected within 300 days. The spinal cord was positioned at different depths within a proton and helium spread-out Bragg peak (SOBP), and either one or two fractions of high doses (up to ~20 Gy) were applied. The endpoint dependent model parameters for this study were derived as $K_{iDSB}$ = 3.5 × 10^−5^ and $K_{cDSB}$ = 9.8 × 10^−3^ via best-fitting (method of least squares) to the reference *TD_50_* photon measurements at a fixed dose rate of 3.75 Gy/min over the number of fractions obtained by Debus et al. [27] and Karger et al. [28] who used the same endpoint (see Supplementary Material Figure S2). The repair half-life times of isolated and clustered damages within UNIVERSE, $T_{iDSB}^{1/2}$ and $T_{cDSB}^{1/2}$, were set to 11.4 and 129.6 min, respectively, by following the findings of Pop et al. [29] (Table 1). In an effort to avoid the exceptionally high computational costs connected with the full calculation of biological effects of an ion beam containing mixed LET and dose rates, the following approximative approach was applied to compare the predictions of the dose-rate effects in UNIVERSE with the measured RBE by Saager et al. [23] and Hintz et al. [24]. In their study, the RBE was determined as the ratio between the reference photon *TD_50_* at 3.75 Gy/min and the *TD_50_* at a given position within the SOBP receiving dose-rate $\dot{D}$:

(1) $$\mathrm{RBE}\;=\;\frac{TD_{50}^{\gamma }\left(3.75\frac{\mathrm{Gy}}{\min}\right)}{TD_{50}^{p}(\dot{D})}\;.$$

**Figure 4:**
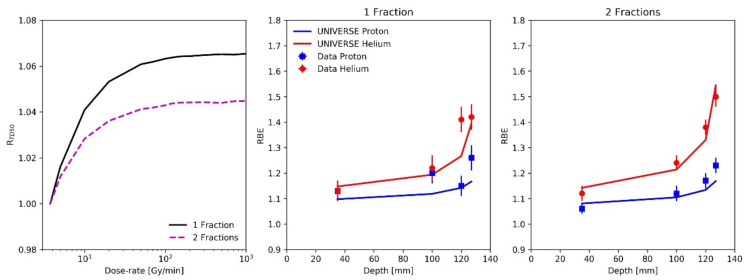
Left panel: The ratio between the *TD_50_* after photon irradiation with a reference dose rate of 3.75 Gy/min and a given dose rate ($R_{TD50}$) as a function of the dose rate for one fraction (black line) and two fractions (dashed purple line). Middle panel and right panel: RBE for *TD_50_* of the rat spinal cord tolerance after application of a proton SOBP (blue squares; taken from Saager et al. [23]) and a helium SOBP (red circles; taken from Hintz et al. [24]) in one and two fractions with corresponding UNIVERSE predictions (solid lines).

However, this expression can be extended to:

(2) $$\mathrm{RBE}\;=\;\frac{TD_{50}^{\gamma }(\dot{D})}{TD_{50}^{p}(\dot{D})}\;\cdot \;\frac{TD_{50}^{\gamma }\left(3.75\frac{\mathrm{Gy}}{\min}\right)}{TD_{50}^{\gamma }(\dot{D})},$$

where the superscripts *γ* and *p* signify the application of photons and (charged) particles, respectively. The first fraction in Equation (2) equals the definition of the dose-rate adapted RBE and the second fraction in Equation (2) describes the relative effectiveness between the reference radiation at the reference dose rate and the dose-rate applied by the ion beam. The latter will be referred to as $R_{TD50}\;:\;=\;TD_{50}^{\gamma }(3.75\;\mathrm{Gy}/\min)/TD_{50}^{\gamma }(\dot{D})$, a value which can be efficiently calculated using the existing dose-rate implementation of UNIVERSE for sparsely ionizing radiation [8] and is depicted over the dose rate in the left panel of Figure 4. While the determination of a mixed field dose-rate adapted RBE remains computationally expensive, the value was found to be well approximated by the no-repair RBE for the dose rates and LET at the investigated positions. Predictions for the no-repair RBE value can also be swiftly calculated for mixed LET fields within our existing modelling framework using pre-processed databases [9]. Taken together, the prediction of the measured dose-rate dependent RBE within the given setup is approximated using the no-repair RBE multiplied by the factor, $R_{TD50}$—and is shown in the middle and right panel of Figure 4 (solid lines):

(3) $$\mathrm{RBE}\;\approx \;\frac{TD_{50}^{\gamma }\left(\infty \right)}{TD_{50}^{p}\left(\infty \right)}\cdot \;R_{TD50}.$$

The calculated dose rate, the corresponding values of $R_{TD50}$, and the simulated dose-weighted LET (LET_d_) for each measurement position of the proton and helium SOBP and fractionation scheme in the studies by Saager et al. [23] and Hintz et al. [24] are shown in Table 3.

**Figure 5:**
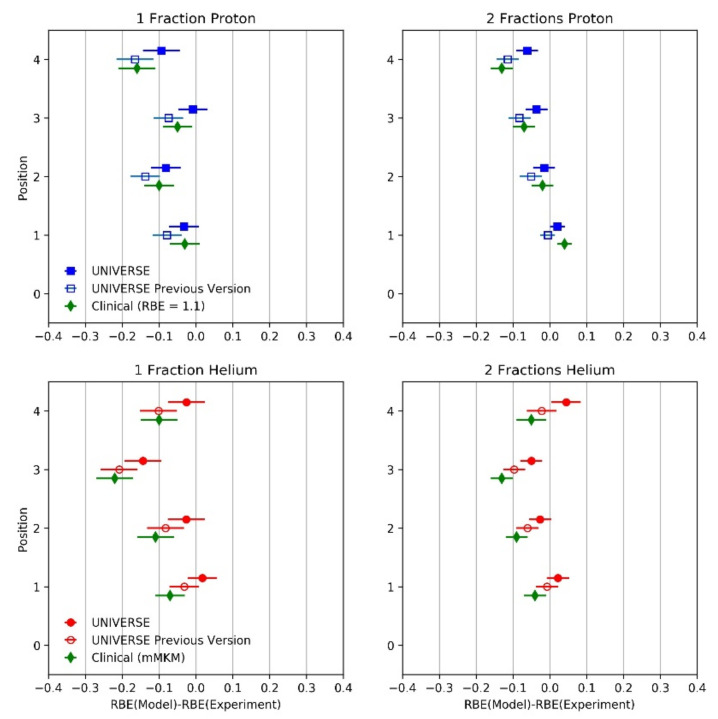
Difference between predicted RBE of *TD_50_* within the rat spinal cord after application of a proton SOBP (**upper** panels; Saager et al. [23]) and a helium SOBP (**lower** panels; taken from Hintz et al. [24]) in one (**left** column) and two fractions (**right** column) and different predictions. UNIVERSE considering (closed blue squares and red circles) and disregarding dose-rate/DNA damage repair (open blue squares and red circles) as well as a fixed RBE of 1.1 for protons and mMKM predictions for helium ions, representing current clinical approaches (green diamonds). Positions 1, 2, 3, and 4 correspond to the depth at 35, 100, 120, and 127 mm, respectively.

To visualize the deviations between the predicted and measured RBE, the difference between the two values is shown in Figure 5 for each measurement position and fractionation for the proton as well as helium SOBP (filled blue squares and red circles). The mean absolute deviations (MAD: the absolute value of the percentage difference between measured and predicted RBE averaged over the values of one particle) for the proton and helium predictions were found to be 3.65% and 3.30%, respectively. As a comparison, the predictions based on the previous version of UNIVERSE as well as the existing clinical approaches are additionally shown. In its previous version, the UNIVERSE prediction would neglect the dose rates applied by the reference radiation and the ion beam and parameters would be derived from reference irradiation data without consideration of its dose rate and its impact on repair processes. Here, they were derived from the same reference *TD_50_* photon measurements as were used earlier (obtained at 3.75 Gy/min) but by applying no DNA damage repair kinetics. Best-fitting (method of least squares; see Supplementary Material Figure S2) yielded $K_{iDSB}$ = 6.3 × 10^−5^ and $K_{cDSB}$ = 8.5 × 10^−3^ (Table 1). Based on these parameters, the RBE predictions were then calculated using UNIVERSE, again without consideration of the DNA repair kinetics (open blue squares and red circles). The MADs for this approach were found to be 7.42% and 5.69% for protons and helium ions, respectively. Representing the current clinical approaches, an RBE value of 1.1 was assumed for the entire proton SOBP and the predictions of the mMKM were computed using the settings applied by Hintz et al. [24] for the helium SOBP (green diamonds). The dose-rate effects were not considered in their study. The MADs of the clinical approaches for protons and helium ions were found to be 6.27% and 8.68%, respectively. In comparison to the clinical approaches, the previous version of UNIVERSE scored an 18% larger MAD for protons and 35% smaller MAD for helium ions. The MADs of the modified version of UNIVERSE were reduced by 51% and 42% for protons and helium ions, respectively, in comparison to the previous version of the model. At the same time, the MADs of the modified UNIVERSE were 42% and 62% smaller than the MADs of the clinical approach for protons and helium ions, respectively.

## 3. Discussion

The observed increase of the fixed-reference RBE with an increasing dose rate and its saturation (Figure 1 and Figure 2) can be readily explained by the fact that with an increasing dose –rate, the irradiation times become gradually shorter in comparison to the repair half-life times of the isolated damages (in our representative analysis: $T_{iDSB}^{1/2}$ = 4 min). This reduces the amount of damage being repaired during irradiation, which in turn increases the lethality of the induced damage pattern. Once the irradiation time is significantly shorter than the repair half-life time, the effect saturates and a further shortening of the irradiation time (i.e., increased dose-rate) has no further effect. Within the dose-rate ranges analyzed in this study, the repair of complex damages plays no significant role as their repair half-life time is often in the order of hours (in our analysis $T_{cDSB}^{1/2}$ = 100 min). This can also explain the slight weakening of the rising trend of the fixed-reference RBE over the dose rate with an increasing LET. At a higher LET, more complex damages are induced and the effectiveness of the ion beam becomes less sensitive to a change in the irradiation time and dose rate. At the same time, even the low LET protons in this study (2 keV/µm) induce slightly higher proportions of complex damages in comparison to the sparsely ionizing reference radiation. Thus, the gain in lethality over the dose rate for the reference radiation is higher than for any of the investigated proton settings, resulting in the observed downward trend of the dose-rate adapted RBE over the dose rate. This effect increases with the difference of the sensitivity to changes in the dose rate between the reference radiation and the ion beam, thereby explaining the greater effect at a higher proton LET—where more complex damages are induced.

The predicted saturation of the biological effect at irradiation timesthat are much shorter than the repair half-life times is consistent with the sparse experimental data available in the literature. Matsuura et al. [12] compared the survival of HSG cells at the plateau (LET_d_: 0.56 keV/µm) and the Bragg peak (LET_d_: 3.19 keV/µm) of a proton beam up to doses of 8 Gy using a conventional dose rate of 8 Gy/min and an increased dose rate of 325 Gy/min, thereby resulting in a maximum irradiation time of 1 min in their study. No significant difference was observed between the survival curves obtained under the two dose-rate settings. Furthermore, Schmid et al. [13] irradiated HeLa cells using a 20MeV proton beam (LET: 2.66 keV/µm) by applying 3 Gy within 100 ms to 1 ns, finding no significant change in the effectiveness of micronuclei production. In both studies, the applied irradiation times were clearly shorter than the typical values of the repair half-life times for isolated damages found in the literature [8,30,31,32], meaning that no differences in RBE would be expected in the given settings. On the other hand, while Bennett et al. [11] applied relatively low doses that are comparable to those in the previously mentioned studies (up to 3 Gy), they observed a decrease in proton RBE at all investigated LET (0.22, 0.74 and 1.26 keV/µm) when the dose rate was reduced. However, the dose-rates applied for this comparison were 0.33 Gy/min and 0.0165 Gy/min, resulting in a maximum irradiation time of 9 min and 3 h, respectively. Especially at the lower dose rate, the irradiation time significantly exceeds common values for the repair half-life time of isolated damages, even reaching common values for complex damages [8,30,31,32] so that significant sensitivity to the changes in the dose rate would be expected. This behavior is also in line with the trends predicted by other biophysical models with a focus on clinical applications, such as the recently published study by Kasamatsu et al. [22], which implies that for significant protractions of the irradiation (i.e., very low dose-rate), a considerable dose-rate effect could even arise at lower clinical doses (~2 Gy). Besides the increasing steepness of the RBE-dose-rate dependency towards a lower dose rate, the growing importance of the dose-rate effect with the applied dose predicted by other models [17,18,19,20] is consistently reflected in our predictions. The range of predicted RBE covered throughout the studied dose-rate range, and concurrently the steepness of the fall off towards low dose rates, visibly increases for both definitions of RBE with the applied dose within our framework (Figure 1 and Figure 2).

Similarly, the discrepancy between the no-repair RBE and the fixed-reference RBE was found to increase with the applied dose and reach significant levels at doses of about 6 Gy and above for the settings chosen in our study (Table 2). The trend can be explained by the following mathematical argument: The fixed-reference RBE is defined as the ratio between the dose applied by the reference radiation (γ) with a (commonly low) fixed dose-rate $\dot{\delta }$ (in our study $\dot{\delta }\;=\;2\;\mathrm{Gy}/\min$), $D_{EL}^{\gamma }(\dot{\delta })$ and the dose applied by the particle beam (*p*) at a given dose-rate $\dot{D}$, $D_{EL}^{p}(\dot{D})$, that leads to the same effect level *EL*: $D_{EL}^{\gamma }(\dot{\delta })/D_{EL}^{p}(\dot{D})$. The maximum (or saturation) value of this RBE definition can be found at the limit of high dose –rates, which can be written as: $D_{EL}^{\gamma }(\dot{\delta })/D_{EL}^{p}\left(\infty \right)$. The no-repair RBE can be interpreted as the same ratio with both doses being applied at infinite dose rates, where repair becomes negligible: $D_{EL}^{\gamma }\left(\infty \right)/D_{EL}^{p}\left(\infty \right)$. Therefore, the maximum ratio between the RBE predicted by both implementations is given by: $D_{EL}^{\gamma }(\dot{\delta })/D_{EL}^{\gamma }\left(\infty \right)$. This again is the definition of the RBE of the reference radiation applied with the infinite dose rate in reference to the same radiation quality applied at a low, fixed dose rate. In our approximative approach to efficiently predict the RBE measurements in the RSC, this factor was termed R_TD50_. It can be shown that within the linear–quadratic model (LQM) [33], the RBE decreases with an increasing dose if and only if [34]:

(4) $$\frac{\alpha_{\infty }}{\alpha_{\dot{\delta }}}>\sqrt{\frac{\beta_{\infty }}{\beta_{\dot{\delta }}}},$$

where $\alpha_{\dot{\delta }}$ and $\beta_{\dot{\delta }}$, as well as $\alpha_{\infty }$ and $\beta_{\infty }$, are the linear and quadratic coefficient of the survival curves resulting from an irradiation with the reference dose rate at the fixed dose rate $\dot{\delta }$ as well as the “infinite” dose rate, respectively. Now, it is well established that survival curves of sparsely ionizing radiation exhibit an increasingly linear behavior with decreasing dose rates [35,36,37], while the value of α remains nearly constant [38]. This means the value of β approaches zero for very low dose rates. In the context of our framework, this can be explained by the effective suppression of cDSB through significant repair during the longer irradiation times. (For illustration purposes, the predicted survival curves of DU145 cells after irradiation with sparsely ionizing radiation at 2 Gy/min and with an “infinite” dose rate are shown in the Supplementary Material). Consequently, the left side of Inequality 4 assumes a value of about one, while the right side easily exceeds this value with $\beta_{\dot{\delta }}$ being significantly smaller than $\beta_{\infty }$, thereby implying the reverse of the inequality to be true. Thus, the ratio $D_{EL}^{\gamma }(\dot{\delta })/D_{EL}^{\gamma }\left(\infty \right)$, and therefore, the relative difference between the no-repair RBE and fixed-reference RBE, increases with the applied dose. The general increase of this discrepancy with LET found in our study can also be understood in this context. The discussed dose increase is equivalent to increasing the effect level at which the two dose rates of the reference radiation are compared to each other. Within the LET range investigated in this study, proton and helium beams generally become more effective with increasing LET, thus leading to a “dose-equivalent” shift of the assessed effect level.

With both our study and the literature pointing towards increased dose-rate effects at high doses, the RBE studies based on rat spinal cords irradiated with high dose proton and helium SOBPs (fractional doses of up to ~20 Gy) by Saager et al. [23] and Hintz et al. [24] appeared to be prime candidates to benchmark our extended model. While biophysical models based on DNA lesions and cell survival might not be necessarily extendable to a model of late tissue toxicity (or any higher level biological endpoint) in a simple and conclusive way, it has become common and accepted practice to extend their prediction by generalizing the meaning of the radiosensitivity parameters to establish the dose-effect relationship and applying the existing framework [23,24,39,40,41,42]. In UNIVERSE, this approach could be motivated by viewing the DNA damage pattern characterized by the amount of isolated and complex DSB as a proxy for the general effect of the applied radiation and coupling it to the investigated endpoint by reinterpreting the parameters $K_{iDSB}$ and $K_{cDSB}$ as probabilities for each of the damage instances to trigger the endpoint of interest. The found values for $K_{iDSB}$ and $K_{cDSB}$ for the in-vivo study are about two orders of magnitude smaller than those used in the in-vitro analysis (Table 1), following a trend already found in an earlier publication [8]. In a study applying the latest version of the “local effect model” (LEM) [41], the authors reduced their radiosensitivity parameters, which coincide with the LQM parameters α and β, by about two orders of magnitude from the values applied for in-vitro predictions for their description of in-vivo endpoints. As a reason for this approach, the authors refer to “… recent findings concerning the observed difference of the absolute LQ-parameters between in-vitro and in-vivo endpoints independent on the tumor type …” [41]. Under given assumptions, the values of $K_{iDSB}$ and $K_{cDSB}$ can be approximated by a linear combination of the LQM parameters [43,44] so that such observations are probably applicable to the UNIVERSE parameters, too. Mechanistically, the lower values of $K_{iDSB}$ and $K_{cDSB}$ could signify the extended effect chain that might lie between the damage pattern within a single cell and the higher-level endpoints that are observed in in-vivo studies. Instead of merely triggering the loss of one colony, a larger number of damaged cells might have to be involved, while at the same time there are potentially several additional steps involved that may need to be taken until the observed endpoint develops. The predictions of the modified UNIVERSE match the trend and values of the data well (middle and right panel Figure 4) and the deviations from the measurements are small (Figure 5). It is noteworthy that there appears to be a significant upward dose-rate gradient within the proton SOBP, while the dose rate within the helium SOBP appears to slightly taper off towards the distal edge (Table 3). This can be explained by the fact that the irradiation field is delivered in subsequent “energy slices” in depth. The dose at the proximal positions is therefore delivered by virtually all energy slices, while the dose at the distal positions is built up by only a few energy slices. However, in the case of the helium beam, a substantial number of secondary particles are formed, which transport parts of the dose along the beam axis towards the distal edge. Thus, at the distal positions in the helium SOBP, the total dose is deposited over a longer period of time, reducing the effective dose rate. Therefore, depending on the irradiation plan and applied particle, different dose-rate profiles may arise, which might need to be considered for an accurate prediction of the RBE. Furthermore, knowledge of the impact of such dose-rate profiles could be important to correctly interpret measured RBE profiles, such as those obtained in the discussed RSC study. Before the modifications presented in this study were implemented in UNIVERSE, the predictions of biological dose in ion beams as well as the model parameters that they were based on were derived with no regard for the dose rate and DNA repair kinetics. Thus, the predictions by this previous version need to be differentiated from the “no-repair RBE” introduced earlier, where the model parameters were derived under the consideration of the dose rate of the reference radiation. The previous approach within UNIVERSE predicts markedly lower RBE values in comparison to the modified version of the model (Figure 5), which can be explained by the neglect for the heightened dose rate within the ion beams in comparison to the reference radiation. While the clinical assumption of an RBE of 1.1 and the previous UNIVERSE approach delivered comparable results, the mMKM predictions for the helium SOBP were found to further deviate from the measured values than the predictions by the former UNIVERSE version. Ultimately, the predictions from the presented modified version of UNIVERSE were not only found to be reduced in comparison to its previous version, but also compared to current clinical approaches. However, the MKM version (mMKM) applied in this study was designed primarily to accommodate a low dose per fraction irradiation planning, disregarding any DNA repair kinetics. A more recent extension of the model, including the repair of sub-lethal damage, could provide improved predictions [17,18,19,20].

Consequently, DNA repair kinetics and the dose rates applied by the reference as well as the ion beam need to be considered for the biophysical models to make precise predictions and allow for the accurate assessment of their benchmarks, as deviations between the prediction and measurement could be misjudged. In practice, this is especially relevant for benchmarks with data from translational in-vivo measurements using endpoints such as paresis after irradiation of the RSC [23,24] (as used in this study) or the regeneration of crypts in mice jejunum [45], which, by design, are needed to assess large doses. In those scenarios, high clinical relevance is paired with an increased potential need to correct for dose-rate effects. Its performance in the benchmark presented has shown the ability for the modified UNIVERSE to provide improved and adequate predictions in such cases. On the other hand, while dependent on the model parameters, our study indicated that substantial deviations between predictions neglecting DNA damage repair (no-repair RBE) and predictions including such mechanisms (fixed-reference RBE) within our model could already arise at doses as low as 6 Gy (Table 2), thereby not only concerning the possible predictions for hypofractionated clinical settings but even higher doses applied in conventional in-vitro measurements. Furthermore, the observed impact of the reference dose rate on the predicted RBE (Figure 3 left panel) does not only underline its importance for accurate predictions by UNIVERSE, but also illustrates the possibly significant impact on the measured RBE. In order to appropriately compare RBE measurements obtained in different setups, one might therefore need to carefully consider the dose rates applied by the respective reference radiations.

The approximation applied in this work to predict the measured RBE in the RSC study enabled a swift calculation and thus avoiding significant computational costs that would have exceeded the scope of this study. The approximation was valid because the value of the dose-rate adapted RBE predicted by the modified implementation was nearly identical to the value predicted by the existing implementation for the dose rates and LET present at the measurement points of the study. However, it would fail as soon as the two values diverge, which would mainly be the case at lower dose rates. Thus, the approximation is not generally applicable, especially in situations including longer irradiation times (e.g., active scanning of large fields, inter-beam irradiation pauses), which would call for the utilization of a full simulation. Further developments are planned to optimize the performance of the model to enable such calculations with UNIVERSE within reasonable computation times.

## 4. Conclusions

The UNIVERSE model of ion beam RBE was extended to consider the time-dependent repair of isolated and complex DNA damages and the resulting effects of the dose rates applied by the reference radiation and the ion beam itself. The predicted trends, including the increased importance of the dose-rate effect with the applied dose and the saturation of effects at high dose rates were found to be consistent with measurements and model-based studies found in the literature. As a benchmark of our developments, clinically relevant RBE measurements by Saager et al. [23] and Hintz et al. [24] in the rat spinal cord irradiated with high dose proton and helium SOBP leading to fields of mixed LET and distinct dose-rate profiles applied in one and two fractions were chosen. The data were matched well by the prediction of the modified UNIVERSE, outperforming its previous version and existing clinical approaches, which neglect dose-rate effects. Our study not only shows that the dose rates applied by the reference as well as the ion beam should be considered for biophysical models to make precise predictions and allow for the accurate assessment of their benchmarks, but also showcases an important step in the development of our framework. UNIVERSE can now better support the correct interpretation of pre-clinical studies by applying high doses and its capabilities to give theoretical guidance during the development of novel techniques in ion beam therapy were extended.

## 5. Materials and Methods

### 5.1. Experimental Data from Literature

The experimental RBE data used to benchmark the model were taken from [23,24].

### 5.2. Modeling Approach

The UNIVERSE is a modelling framework developed to describe the effects of ionizing radiation in biological systems considering numerous factors, including the effect of interference with the DNA damage repair process [6,9], radical scavengers [7], and the applied dose rate [8,10]. For this study, the implementation of DNA repair kinetics and that of ion beams from two recent publications [8,9] are combined to study the dose-rate effects in ion beams. For detailed descriptions of the implementation and parameters of the individual sub-components, we kindly refer to the respective references. Here, a basic description of the concepts relevant to this study shall be given.

In UNIVERSE, the survival probability of a cell is determined by the amount and distribution of DNA double strand breaks (DSB). More specifically, the chromatin is supposed to be divided into domains containing about 2 Mbp of DNA, resembling chromatin substructures referred to as “giant-loops” [46,47,48,49,50]. A domain containing only a single DSB is classified as an “isolated” DSB (*iDSB*), while those containing two or more are labeled as “complex” or “clustered” DSB (*cDSB*), thereby following the approach of others [43,51]. This classification was found to resemble the populations of quickly and slowly repaired DSB after irradiation [32,52]. Based on the number of isolated DSB ($N_{iDSB}$) and complex DSB ($N_{cDSB}$) within the nucleus, and given the probability that one *iDSB* or one *cDSB* inactivates the cells described by the cell-line dependent lethality parameters $K_{iDSB}$ and $K_{cDSB}$, respectively, one can determined the cell survival probability (S) using [6,43]:

(5) $$S\;=\;{\left(1-K_{iDSB}\right)}^{N_{iDSB}}\cdot {\left(1-K_{cDSB}\right)}^{N_{cDSB}}.$$

For sparsely ionizing radiation, such as photons and most electron beams, the deposited dose is thought to be homogenously spread throughout the cell’s nucleus. With an average DSB yield of $\alpha_{DSB}\;=\;30\frac{\mathrm{DSB}}{\mathrm{Gy}\cdot \mathrm{Cell}}$ [53], which is found to be constant within clinical dose ranges [54], the expected number of induced DSB after irradiation with a dose *D* can be written as $\langle N_{tDSB}\rangle \;=\;\alpha_{DSB}\cdot D$. The survival fraction within an irradiated cell population can then be predicted as follows: The induced number of DSB is sampled from a Poisson distribution based on its expectation value for >10^4^ iterations. For each iteration, each induced DSB is randomly assigned to a domain within the nucleus with a uniform probability. Ultimately, the number of domains containing a single DSB (*iDSB*) and two or more DSB (*cDSB*) are scored, and the survival probability is determined using Equation (5). Finally, these results are averaged over all iterations.

In contrast, for ion beams, the heterogenous dose deposition by single ion traversals and their radial dose distribution (RDD) needs to be considered in a geometrical representation of the cell nucleus. In our implementation [9], the nuclear geometry is approximated by a cylinder containing cubic domains, through which the particles pass parallel to its height-axis. The RDD of the tracks are described with the Kiefer–Chatterjee parametrization [55,56] and consists of an inner “core” region with constant dose $D_{c}$ and a radius of $r_{min}$ as well as an outer “penumbra” region covering a radius of $r_{max}$ and a dose of $D_{p}$, which decreases as a function of the distance to the track center r (here given in µm) [57,58]:

(6) $$D_{c}\;=\;\frac{1}{\pi \;r_{min}^{2}}\;\left(\frac{LET}{\rho }-2\;\pi \;K_{p}\;ln\left(\frac{r_{max}}{r_{min}}\right)\right),$$

(7) $$D_{p}\left(r\right)\;=\;K_{p}\;r^{-2},$$

(8) $$K_{p}\;=\;1.25\cdot 10^{-4}\;{\left(\frac{z^{*}}{\beta_{ion}}\right)}^{2},$$

where LET is the unrestricted linear energy transfer of the particle in keV/µm, ρ is the density of water in $g/{\mathrm{cm}}^{3}$, $\beta_{ion}\;=\;\frac{v}{c}$ (v: particle velocity, c: speed of light), and $z^{*}$ is the effective charge of the particle, given by [59]:

(9) $$z^{*}\;=\;z_{ion}\left(1-\exp\left(-125\;\beta_{ion}\;z_{ion}^{-2/3}\right)\right),$$

with the charge of the fully ionized particle $z_{ion}$. The core radius is dependent on the velocity of the particle and can be determined by $r_{min}\;=\;\beta_{ion}\cdot r_{c}$, where $r_{c}$ was derived to be 11.6 nm [55,58]. Furthermore, the maximum extent of the RDD ($r_{max}$) increases with the kinetic energy of the particle $E_{kin}$ and can be estimated (in µm) as $r_{max}\;=\;\epsilon \cdot E_{kin}^{\delta }$, with $E_{kin}$ in units of MeV/u, ϵ = 0.062, and δ = 1.7 [56,58]. The diffusion of radical species produced within the initial RDD ($D\left(r\right)$) was considered by convolution with a normal distribution kernel with a standard deviation σ, which characterizes the diffusion length of the radicals. The resulting diffused RDD ($\tilde{D}\left(r\right)$) is then given by [60,61]:

(10) $$\tilde{D}\left(r\right)\;=\;\frac{e^{-r^{2}/2\sigma^{2}}}{\sigma^{2}}\int_{0}^{\infty }dr^{\prime }r^{\prime }e^{-\frac{r^{\prime 2}}{2\sigma^{2}}}\;I_{0}\left(\frac{r\;r^{\prime }}{\sigma^{2}}\right)D\left(r^{\prime }\right),$$

with the modified Bessel function of order zero $I_{0}$. To improve the performance of UNIVERSE simulations on Graphics Processing Units (GPU), the diffused RDD was parametrized with an approximative three-step function. Based on the assumed radius of the nucleus and the maximum extension of the penumbra, the area through which a tracks core needs to pass in order to deposit any dose in the nucleus can be determined. Using the LET of the particle and the macroscopic planned dose, the expected number of tracks passing this area is computed. For each iteration, the number of tracks that deposit a dose in the nucleus is sampled from a Poisson distribution using the previously calculated expectancy value. These tracks are randomly assigned a position within the aforementioned area following a uniform distribution. The dose deposited by each track traversal within each nuclear domain can be computed using this RDD parametrization, thereby resulting in an expectancy value for an induced DSB in the individual domains, which are again used to sample the actual amount of DSB in each domain following a Poisson distribution. The high local doses (>>100 Gy), which can arise within a charged particle track, are believed to result in the aggregation of DNA single strand breaks on opposite strands of the DNA, ultimately resulting in the formation of DSB and an increase in the expected number of DSB within the affected domains. In our implementation, this process is considered using an analytical formula proposed by Friedrich et al. [62], which effectively results in an increased yield of DSB with LET. Following the hypothesis that the survival probability of a cell is only dependent on the induced damage and not the radiation quality that caused it, the amount of isolated as well as complex DSB and the resulting survival probability is determined for individual iterations following Equation (5) and using the same lethality parameters, $K_{iDSB}$ and $K_{cDSB}$, as for sparsely ionizing radiation. The results are again averaged over all iterations to yield the prediction for the surviving fraction of the cell population after irradiation. Thus, the cell-line dependent parameters $K_{iDSB}$ and $K_{cDSB}$ are usually obtained by fitting the model for sparsely ionizing radiation to the corresponding data and subsequently used to predict the effect of ion beams.

When the repair kinetics of the induced DSB and the impact of the applied dose rate is considered within UNIVERSE, the total irradiation time of a simulated setup is divided into a number of time-steps ($N_{t}$ = 100). For both sparsely ionizing radiation and ion beams, the distribution of DSB within the domains is simulated for partial doses $D_{part}\;=\;D/N_{t}$ and added to the existing DSB distribution at each time step. For each iDSB and cDSB, a lifetime is randomly sampled from an exponential distribution characterized by a shorter and longer repair half-life time, $T_{iDSB}^{1/2}$ and $T_{cDSB}^{1/2}$, respectively. These cell-line dependent values can be adopted from the results of DSB re-joining studies in the literature or are fitted to data. If at any timestep any DSB is added to a domain classified as an iDSB, it is transformed into a cDSB and the lifetime of this damage is redrawn on the basis of $T_{cDSB}^{1/2}$. At each timestep, the sampled lifetimes of the DSB are checked and any damage exceeding its lifetime is repaired (i.e., removed from the domain). Each instance of repair however has a chance to fail with the probability of $K_{iDSB}$ and $K_{cDSB}$ for iDSB and cDSB, respectively. In case of such a “misrepair” event, the cell is believed to be inactivated and its survival probability is defined as zero, through which the consistency of the algorithm is ensured when repair processes are considered over different periods of time. For each iteration, the survival probability is again calculated using Equation (5) at the end of the irradiation time. If no misrepair event occurred, the average value of all iterations is used as the survival fraction after irradiation with a given dose rate.

### 5.3. RBE Definitions

The fixed-reference RBE is defined as the ratio between the dose applied by the reference radiation (γ) with a (commonly low) fixed dose rate $\dot{\delta }$ (in our representative study $\dot{\delta }\;=\;2\;\mathrm{Gy}/\min$), $D_{EL}^{\gamma }(\dot{\delta })$, and the dose applied by the particle beam (*p*) at a given dose rate $\dot{D}$, $D_{EL}^{p}(\dot{D})$ leads to the same effect level *EL*:

(11) $$\mathrm{Fixed}\text{-}\mathrm{Reference}\;\mathrm{RBE}\;=\;D_{EL}^{\gamma }(\dot{\delta })/D_{EL}^{p}(\dot{D}).$$

In most RBE studies and measurements, the dose rate of the reference radiation is a constant value.

In the case of the dose-rate adapted RBE, the dose rate of the reference radiation is adapted to the dose rate $\dot{D}$ applied by the particle beam so that its definition reads:

(12) $$\mathrm{Dose}\text{-}\mathrm{Rate}\;\mathrm{Adapted}\;\mathrm{RBE}\;=\;D_{EL}^{\gamma }(\dot{D})/D_{EL}^{p}(\dot{D}).\;$$

This definition is an approach to describe the “intrinsic” efficacy of the applied ion beam in comparison to the reference radiation for a certain cell-line at a given dose rate.

As the irradiation time approaches zero for extremely high dose rates, the RBE, under the assumption of no repair taking place, can also be seen as the RBE that is expected if both the reference radiation and the ion beam were to be applied at infinite dose rates:

(13) $$\mathrm{No}\text{-}\mathrm{Repair}\;\mathrm{RBE}\;=\;D_{EL}^{\gamma }\left(\infty \right)/D_{EL}^{p}\left(\infty \right).$$

### 5.4. Monte Carlo Simulation of Dose, Dose-Rate, and Rat Spinal Cord RBE in Spread-Out Bragg Peak

The 6 cm deep proton and helium SOBPs centered at 10 cm depth applied by Saager et al. [23] and Hintz et al. [24] were computed via the FLUKA Monte Carlo simulation [63,64] using beam delivery records and log-files. For this, a complete geometry of the HIT beamline was used [65]. The physical dose, biological dose, and dose rate were simulated for the four experimental positions: 35 mm, 100 mm, 120 mm, and 127 mm. The biological dose was calculated using approaches detailed elsewhere [9,66], including the contributions of secondary particles. The proton (helium) irradiation plan consisted of 29 (31) energy slices separated by ~3.5 s spill-to-spill time with a nominal spill intensity of 3.2 × 10^9^ (8 × 10^8^) particle/s. In order to calculate the mean dose rate at each measurement depth, the total time needed to deliver each *TD_50_* dose at four depths were stored during the simulation. Then, for each depth, the mean dose rate was calculated as the *TD_50_* dose divided by this time.

## Figures and Tables

**Table 2:** Maximum relative difference between fixed-reference RBE and no-repair RBE for the analysis shown in Figure 1 and Figure 2. The maximum relative difference was determined at the highest dose rate analyzed (saturation value).

Dose	2 keV/μm	8 keV/μm	25 keV/μm
2 Gy	1.3%	1.8%	3.5%
6 Gy	6.2%	5.1%	9.9%
12 Gy	12.9%	16.6%	22.2%
24 Gy	34.1%	36.8%	45.4%

**Table 3:** Calculated dose rate, corresponding values of $R_{TD50}$, and simulated dose-weighted LET (LET_d_) for each measurement position of the proton and helium SOBP and fractionation scheme in the studies of Saager et al. [23] and Hintz et al. [24].

Particle (No. of Fractions)	Depth [mm]	Dose-Rate [Gy/min]	*R*_*TD*50_	LET_d_ [keV/µm]
Proton (1 Fraction)	35	11	1.042	2.0
	100	18	1.051	3.0
	120	42	1.059	4.1
	127	53	1.061	5.3
Proton (2 Fractions)	35	8	1.022	2.0
	100	14	1.031	3.0
	120	31	1.038	4.1
	127	41	1.040	5.3
Helium (1 Fraction)	35	11	1.042	4.2
	100	11	1.042	9.3
	120	10	1.041	14.4
	127	9	1.036	22.0
Helium (2 Fractions)	35	8	1.022	4.2
	100	7	1.018	9.3
	120	7	1.018	14.4
	127	6	1.015	22.0

## Data Availability

Not applicable.

## Supplementary Materials

The following supporting information can be downloaded at: https://www.mdpi.com/article/10.3390/ijms23116268/s1.
