# Supplementary material for: Impact of DNA Repair Kinetics and Dose Rate on RBE Predictions in the UNIVERSE
Source: Int J Mol Sci. 2022 Jun 3;23(11):6268. doi: 10.3390/ijms23116268 (PMC9181644; doi:10.3390/ijms23116268)
Supplement: Supplementary file 1 [file ijms-23-06268-s001.zip › ijms-1703646-supplementary.pdf]

Supplementary Material

RBE predictions over the applied Dose-Rate for Helium Ions

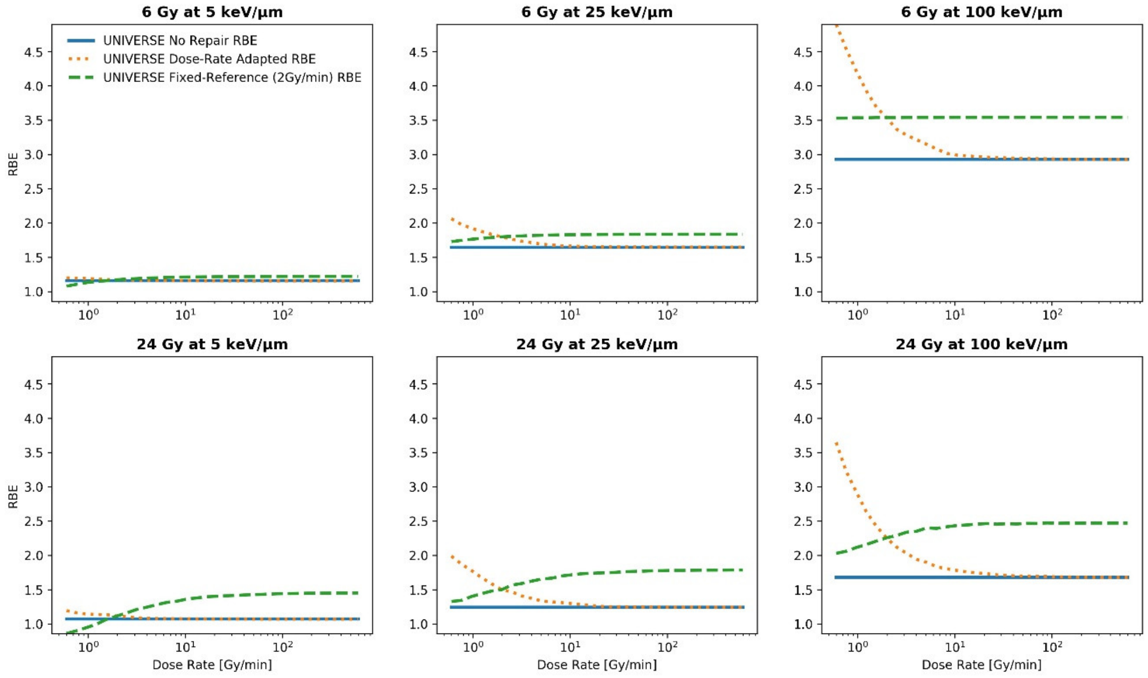

Figure S1. Predictions for the DU145 cell-line over the applied dose-rate for the fixed-reference RBE (2 Gy/min; green dashed line) as well as the dose-rate adapted RBE (dotted orange line) using the extended version of the UNIVERSE for three representative LET values at 6 Gy and 24 Gy. The no-repair RBE is shown for reference (solid blue line). The trends described for the same predictions for protons in the main text are also valid for helium ions for the applied settings.

Photon TD_{50} over Number of applied Fractions

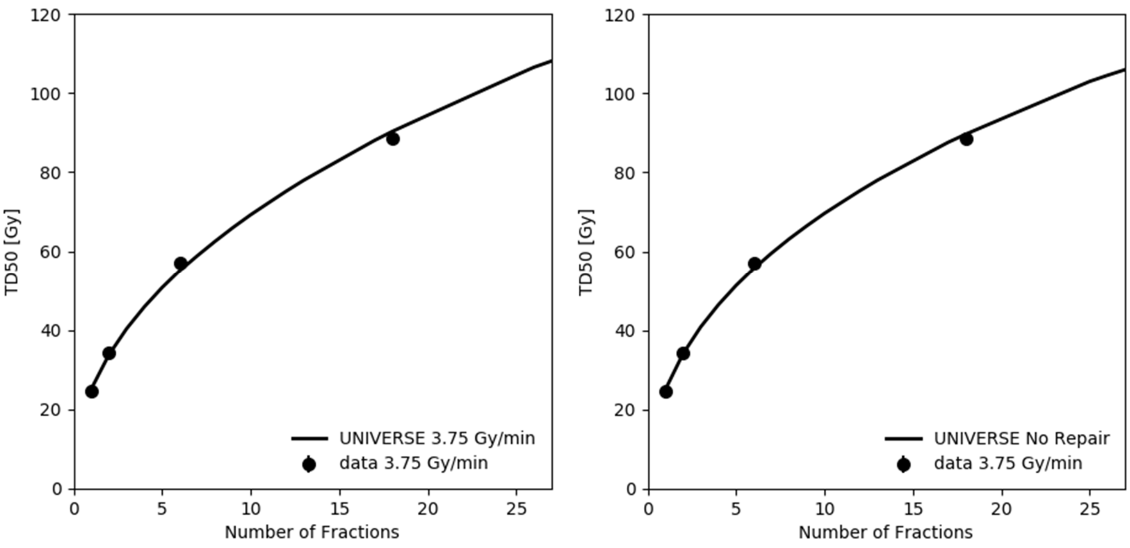

Figure S2. The TD_{50} values measured by Debus et al. [1] and Karger et al. [2] with photon beams for the same endpoint as the rat spinal cord study analyzed in the main text was used to derive the endpoint dependent model parameters K_{iDSB} and K_{cDSB} by fitting UNIVERSE to the data using the least squares method. When the applied dose-rate of 3.75 Gy/min and the resulting repair was considered (left panel) values of K_{iDSB}=3.5x10^{-5} and K_{cDSB}=9.8x10^{-3} were found. Disregarding any repair mechanisms (right panel) yielded K_{iDSB}=6.3x10^{-5} and K_{cDSB}=8.5x10^{-3}.

Survival Curves at low and high Dose-Rates

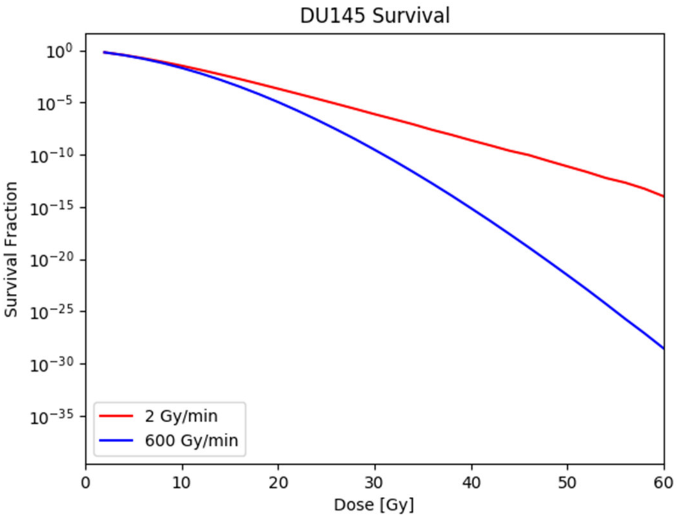

Figure S3. UNIVERSE predictions of the DU145 cell survival curves at a low dose-rate (2 Gy/min; red line) and very high dose-rate (600 Gy/min; blue line). For the high dose-rate repair processes are insignificant (irradiation time of 6 seconds for 60 Gy).

References Supplementary Material

1. Debus, J.; Scholz, M.; Haberer, T.; Peschke, P.; Jäkel, O.; Karger, C.P.; Wannenmacher, M. Radiation Tolerance of the Rat Spinal Cord after Single and Split Doses of Photons and Carbon Ions. *Radiat. Res.* **2003**, *160*, 536–542.
2. Karger, C.P.; Peschke, P.; Sanchez-Brandelik, R.; Scholz, M.; Debus, J. Radiation Tolerance of the Rat Spinal Cord after 6 and 18 Fractions of Photons and Carbon Ions: Experimental Results and Clinical Implications. *Int. J. Radiat. Oncol. Biol. Phys.* **2006**, *66*, 1488–1497, doi:10.1016/j.ijrobp.2006.08.045.
